# Acute myocardial infarction does not affect functional characteristics of adipose-derived stem cells in rats, but reduces the number of stem cells in adipose tissue

**DOI:** 10.1007/s00441-015-2239-z

**Published:** 2015-07-23

**Authors:** B. A. Naaijkens, P. A. J. Krijnen, E. Meinster, E. N. ter Horst, K. Vo, R. J. P. Musters, O. Kamp, H. W. M. Niessen, L. J. M. Juffermans, A. van Dijk

**Affiliations:** Department of Pathology, VU University Medical Center, De Boelelaan 1117, 1081 HV Amsterdam, Netherlands; Department of Cardiac Surgery, VU University Medical Center, Amsterdam, Netherlands; Department of Physiology, VU University Medical Center, Amsterdam, Netherlands; Department of Cardiology, VU University Medical Center, Amsterdam, Netherlands; Interuniversity Cardiology Institute of the Netherlands (ICIN), Utrecht, Netherlands; Institute of Cardiovascular Research (ICaR-VU), VU University Medical Center, Amsterdam, Netherlands

**Keywords:** Adipose-derived stem cells, Acute myocardial infarction, Stromal vascular fraction, Mesenchymal stem cells, Rat acute myocardial infarction model

## Abstract

In most pre-clinical animal studies investigating stem cell therapy in acute myocardial infarction (AMI), the administered stem cells are isolated from healthy donors. In clinical practice, however, patients who suffer from AMI will receive autologous cells, for example using adipose-derived stem cells (ASC). During AMI, inflammation is induced and we hypothesized that this might affect characteristics of ASC. To investigate this, ASC were isolated from rat adipose tissue 1 day (1D group, *n* = 5) or 7 days (7D group, *n* = 6) post-AMI, and were compared with ASC from healthy control rats (Control group, *n* = 6) and sham-operated rats (Sham 1D group, *n* = 5). We found that significantly fewer ASC were present 1 day post-AMI in the stromal vascular fraction (SVF), determined by a colony-forming-unit assay (*p* < 0.001 vs. Control and 7D). These data were confirmed by flow cytometry, showing fewer CD90-positive cells in SVF of the 1D group. When cultured, no differences were found in proliferation rate and cell size between the groups in the first three passages. Also, no difference in the differentiation capacity of ASC was found. In conclusion, it was shown that significantly fewer stem cells were present in the SVF 1 day post-AMI; however, the stem cells that were present showed no functional differences.

## Introduction

Cardiovascular disease is still one of the main causes of mortality and morbidity in the western world, with acute myocardial infarction (AMI) as the most common cardiovascular entity (Shah et al. 2011; Roger et al. 2012; Kuraitis et al. 2011). Although life-saving treatments, such as pharmacotherapeutics and percutaneous coronary intervention, have drastically improved survival rate after AMI, these therapies do not restore damaged myocardial tissue (Roger et al. 2012; Kuraitis et al. 2011; Pfeffer and Braunwald 1990). Regeneration of the myocardium using adult mesenchymal stem cells, however, is a promising solution to restore myocardium function and prevent myocardium failure. This effect may either be reached by differentiation of stem cells towards cardiomyocytes (Naaijkens et al. 2012) or via the secretion of beneficial growth factors and cytokines, the so-called paracrine effect (Boyle et al. 2011; Li et al. 2009). In addition, it is possible to treat patients with their own stem cells, thereby preventing a specific immune response to the administered stem cells (Boyle et al. 2011; Pittenger et al. 2004). In the previous decade, adult mesenchymal stem cells were commonly isolated from bone marrow (bone marrow mesenchymal stem cells; BM-MSC) (Friedenstein et al. 1974; Prins et al. 2009). Recently, adipose tissue has become an alternative, more easily accessible source, providing more mesenchymal stem cells per gram of tissue with a less painful isolation procedure compared to a bone-marrow puncture. By enzymatic digestion of adipose tissue, the stromal vascular fraction (SVF) can be obtained. This is a heterogeneous population of cells and contains amongst others endothelial cells, leukocytes, fibroblasts, macrophages and ASC (Zuk et al. 2001; Bourin et al. 2013). After seeding the SVF in tissue culture plates using specific stem cell proliferation medium, the adherent cell fraction consists almost solely of ASC (Zuk et al. 2001; Bourin et al. 2013). ASC have been studied extensively in pre-clinical AMI models, where they improved heart function and reduced infarct size (Yamada et al. 2006; Mazo et al. 2012; van Dijk et al. 2011).

Although BM-MSC therapy has proven to be safe in clinical applications, it is not yet as beneficial in AMI patients as in pre-clinical studies (Schachinger et al. 2006; Wollert et al. 2004; Janssens et al. 2006). Recently, the first clinical trial using autologous ASC as cellular therapy after AMI (APOLLO trial) only showed minor beneficial effects on myocardial function (Houtgraaf et al. 2012). In all pre-clinical studies that investigated stem cell therapy after AMI, however, the administered stem cells were isolated from healthy donors. In clinical practice, however, patients that suffered from AMI receive their own stem cells. Importantly, during AMI, many inflammatory mediators are released into the blood stream (Kempf et al. 2006). In a recent in vitro study, it was demonstrated that one of these mediators, TNFα, can stimulate BM-MSC to activate macrophages to release the anti-inflammatory interleukin-10 (Nemeth et al. 2009). Conversely, it has been shown that TNFα can negatively influence differentiation of BM-MSC (Relic et al. 2006). We hypothesized that the systemic inflammation caused by AMI might adversely affect ASC within the highly vascularized adipose tissue.

Therefore, we investigated whether stem cells isolated from the inguinal fat pad of rats that suffered a temporal ligation of the left anterior descending coronary artery, were different from stem cells isolated from healthy control rats. For this, we analyzed the percentage of ASC within the SVF, and studied expansion rate and differentiation capacity.

## Materials and methods

### Induction of infarction in rats

Animals were treated according to national guidelines and with permission of the Institutional Animal Care and local Animal Ethical Committee of the VU University Medical Center (Amsterdam, The Netherlands), which conforms with the National Institutes of Health *Guide for the Care and Use of Laboratory Animals* (NIH Pub. No. 85–23, Revised 1996). Male Wistar rats (Harlan Laboratories, Horst, The Netherlands; 300–400 g) were housed under constant temperature (21–22 °C), humidity (60–65 %) and light–dark periodicity (L:D 12:12; lights on from 0700 to 1900 hours). Experimental procedures, started after 2 weeks of acclimatization, were performed by an experienced researcher and previously described in detail (Naaijkens et al. 2014). In short, rats were anaesthetized using subcutaneous hypnorm/dormicum (fetanyl and fluanisone 0.5 ml/kg, midazolam 5 mg/kg) injection and ventilated at 75 breaths/min, 10–0.4 mbar (Zoovent ventilator, The Netherlands). In an additional series of experiments, animals were sham-operated (Sham group) or not operated (healthy control). These rats were anaesthesized using sufentanil (50 μg/kg) and medetomidine (150 μl/kg) subcutaneously. Because these experiments had to be performed under different aneasthesia, as hypnorm/dormicum was no longer available, an additional healthy control group was also included. Data from the sham-operated rats can therefore only be compared with the second Control group (named ‘non-operated control group’). These data are described in the “Results”, but not shown in the graphs. Heart rate was monitored using an Einthoven I ECG. A left thoracotomy was performed between the fourth and fifth rib. Subsequently, a 6.0 prolene suture (Ethicon, Germany) was placed around the left anterior descending coronary artery in 12 rats. Ischemia was maintained for 40 min, followed by reperfusion and chest closure. One rat died during induction of the AMI and was excluded from the study. Rats were sacrificed 1 day (1D group, *n* = 5) or 7 days (7D group, *n* = 6) post-AMI. Healthy control rats (Control group, *n* = 6) were not operated on. Additional sham-operated rats (Sham group, *n* = 5) were operated and the suture was placed, but not tightened, and no infarction was induced. Sham-operated rats were sacrificed 1 day post-operation.

### Analysis of infarction area

Immediately after sacrificing the rats, the hearts were excised and the part of the heart below the suture was cut into 3 equal cross-sections (approximately 2 mm thick). These cross-sections were then snap-frozen in liquid nitrogen. For analysis of the infarcted area, 4-μm cryostat tissue sections were cut from each of the heart cross-sections and transferred onto superfrost tissue slides. The slides were fixed in 100 % acetone for 10 min and washed five times with PBS. Next, the slides were incubated in Bouin at 60 °C for 30 min, cooled for 15 min, and then washed in water for 10 min. The slides were incubated in phosphotungstic acid haematoxylin (PTAH) at 60 °C for 30 min. After cooling, they were dehydrated, washed in xylene and covered. The slides were scanned (Zeiss light microscope) and the surface areas of the total heart cross-section and of the infarcted area were measured using ImageJ software. For the three cross-sections, the infarcted areas were determined as the percentage of the respective surface areas of the total cross-section surface areas and then averaged to produce the final infarcted areas.

### Isolation of the SVF from rat adipose tissue and culture of ASC

Directly after sacrifice, adipose tissue from the inguinal fat pad of male Wistar rats (Harlan) was resected, transferred to sterile PBS and processed immediately, as previously described by van Dijk et al. (2011). The isolation of the SVF cells was performed by an experienced researcher, and rats of the different groups were randomized over different isolation days. In short, adipose tissue was minced and washed with PBS. Then, the extracellular matrix was enzymatically digested with 0.0125 % Liberase™ Research Grade medium Thermolysin (Roche Diagnostics, Indianapolis, USA) under continuous shaking at 37 °C for 25 min. The mixture was filtered (100 μm; Codan, Germany) and centrifuged (5 min, 600*g*). The supernatant was discarded and the cell-containing pellet was resuspended in PBS and centrifuged (5 min, 600*g*). The total number of cells (stromal vascular fraction; SVF) was counted, and either frozen in Recovery cell culture freezing medium (Gibco, Invitrogen, CA, USA) and stored in liquid nitrogen, or directly used in experiments.

### Colony-forming-unit assay

To assess the percentage of colony-forming cells present in the SVF, a colony-forming-unit assay was performed, which is an assay to determine the percentage of ASC present in the SVF (Bourin et al. 2013). For this, SVF cells were seeded at a density of 10 and 100 cells/cm^2^ (in triplicate) and cultured in normal proliferation medium containing DMEM medium, supplemented with 10 % fetal bovine serum (FBS), 1 % penicillin/streptomycin and 1 % L-glutamine (all from Gibco). Cells were maintained at 37 °C, 95 % humidity and 5 % CO_2_. After 14 days, the cells were washed with PBS, fixed with 4 % formalin for 10 min, and subsequently stained in a 1 % toluidine blue solution in borax buffer for 1 min. After washing with water, colonies of approximately 50 cells or more were scored using a stereomicroscope (Zeiss, Germany).

### ASC culture

For culturing of ASC, the SVF was seeded at 100,000 cells/cm^2^ in 25 cm^2^ tissue culture plastic and cultured in normal proliferation medium. Cells were counted after 4 days to determine the number of cells, or grown to near confluency (90 %) and used in further experiments. ASC were detached with 0.5 mM EDTA/0.05 % trypsin (Gibco). Cell size was determined using a Scepter handheld automatic cell counter (Millipore, Billerica, MA, USA). Population doubling time was determined by seeding 2500 cells/cm^2^ (passage 1) in duplicate. When cells reached 90 % confluence, they were harvested and counted with the Scepter handheld automatic cell counter (Millipore). Population doubling time was calculated by dividing the number of days before passaging by the number of cell doublings. This was repeated for passages 2 and 3.

### Flow cytometry

Freshly isolated SVF and cultured ASC were phenotypically characterized using fluorescence-activated cell sorting (FACS; FACS Calibur, Becton Dickinson, USA). Cells were harvested and washed with FACS buffer (PBS containing 1 % BSA and 0.05 % sodium azide). Thereafter, cells were incubated with the appropriate antibodies in FACS buffer at RT for 30 min. The following primary antibodies were used: phycoerythrin (PE)-conjugated mouse-α-rat antibodies against CD90 (1:20; Cedarlane Laboratories, Ontario, Canada), CD45 (1:20; BD Biosciences, San José, CA, USA), CD271 (1:25; BD Biosciences) and CD31 (1:20; AbD Serotec, Oxford, UK), PE-labeled mouse α-human antibody CD105 (1:25; Caltag, Invitrogen, USA), as well as unlabeled mouse-α-rat antibodies against CD34 (1:20; Santa Cruz Biotechnology) and CD73 (1:20; BD Biosciences). After incubation, the cells were washed with FACS buffer. For CD34 and CD73, a fluorescein isothiocyanate (FITC)-conjugated goat-α-mouse secondary antibody was used (1:30; BD Biosciences). After a 30-min incubation period at RT, cells were washed with FACS buffer, centrifuged and resuspended in 100 μl FACS buffer. Isotype control PE- and FITC-conjugated IgG1 antibodies (both BD Biosciences) were used to determine nonspecific fluorescence. Data were analyzed using CellQuest-Pro software and expressed as percentage of positive cells.

### Osteogenic differentiation

To assess the differentiation capacity of ASC towards osteoblasts, ASC (passage 1) were seeded in 12-well culture dishes at 10,000 cells/cm^2^. The cells were then cultured in monolayer in Osteogenesis Differentiation Medium (Gibco). Control cells were cultured in normal culture medium. Both differentiation and control media were changed twice a week. Differentiation was stopped at 12 days by fixating the cells with 4 % formaldehyde at RT for 10 min. Differentiation was demonstrated by staining alkaline phosphatase (ALP). For this, cells were incubated in 0.2 M TRIS-hydrochloride (pH 10), 0.2 M calcium, 0.1 M magnesium chloride solution for 10 min, whereafter a solution containing 0.2 M TRIS-hydrochloride (pH 10), 0.2 M calcium chloride, 0.1 M magnesium chloride solution and nitroblue tetrazolium/5-bromo-4-chloro-3-indolyl-phosphate (NBT/BCIP) was added (1:100, Roche Diagnostics, Mannheim, Germany) for 15 min. This stains ALP blue. Microscopic images were taken using a Zeiss microscope.

### Adipogenic differentiation

To assess the differentiation capacity of ASC towards adipocytes, ASC (passage 1) were seeded in 12-well culture dishes at 5000 cells/cm^2^. The cells were then cultured in monolayer in Adipogenic Differentiation Medium (Gibco). Control cells were cultured in normal culture medium. Both differentiation and control media were changed twice a week. Differentiation was stopped at day 7 by fixating the cells with 4 % formaldehyde for 10 min and with Baker’s calcium formalin for 60 min, both at RT. Subsequently, the cells were washed with isopropanol 60 %, incubated with a filtered Oil Red-O solution (15 min at RT), and counterstained with hematoxylin (5 min at RT). Lipid vesicles, which are a characteristic of adipocytes, were stained in red. Microscopic images were taken using a Zeiss microscope.

### Cardiomyocyte differentiation

To assess the differentiation capacity of ASC towards cardiomyocytes, ASC (passage 1) were seeded in a density of 5000 cells/cm^2^ on laminin-coated (0.2 μg/cm^2^) tissue culture plastic. Cells were stimulated with 5-aza-2-deoxycytidine (9 μM; Fluka, Sigma Aldrich, St. Louis, MO, USA) for 24 h in DMEM supplemented with 15 % FBS, 100 U/ml penicillin, 100 μg/ml streptomycin, 2 mM L-glutamine and 1 % ITS + premix (BD Biosciences). Control cells were cultured in normal culture medium. Both cardiomyogenic and control media were changed twice a week. Cells were harvested after 12 days. Cytospin slides were prepared by spinning ASC onto glass microscope slides at 500 rpm for 5 min (Shandon cytospin 3; Thermo Scientific, Waltham, MA, USA). Slides were air-dried overnight, fixed with acetone for 10 min, and incubated with a rabbit-α-rat connexin 43 antibody (1:1000; Invitrogen) or with a rabbit-anti-rat cardiac Troponin I (cTnI) antibody (1:250; Abcam) in Antibody Diluent (ImmunoLogic, Duiven, The Netherlands) at RT for 60 min. As secondary antibody, a biotin-labeled α-rabbit antibody (1:100) was used for both stainings, followed by incubation of streptavidin/HRP (1:200; both DakoCytomation, Glostrup, Denmark), both at RT for 30 min. Slides were washed with PBS after each step. Stainings were visualized using 5-min incubation with aminoethylcarbazole (AEC; Zymed AEC kit, Invitrogen). Finally, cells were counterstained with hematoxylin and covered. PBS control slides were incubated with PBS instead of the primary antibody and showed no positive staining. Microscopic images were taken using a Zeiss microscope and all cytospin slides were scored for the percentage of connexin 43 and cTnI-positive cells.

### Statistical analysis

Statistical analysis was performed with GraphPad Prism 5. Groups were tested for normal distribution with the one-sample Kolmogorov–Smirnov test. A Student’s *t* test or ANOVA with the Bonferroni post hoc test was used, since all values were distributed normally. A *p* value smaller than 0.05 was considered to represent a statistically significant difference. Data are presented as mean ± standard deviation.

## Results

### Induction of an acute myocardial infarction

**Fig. 1:**
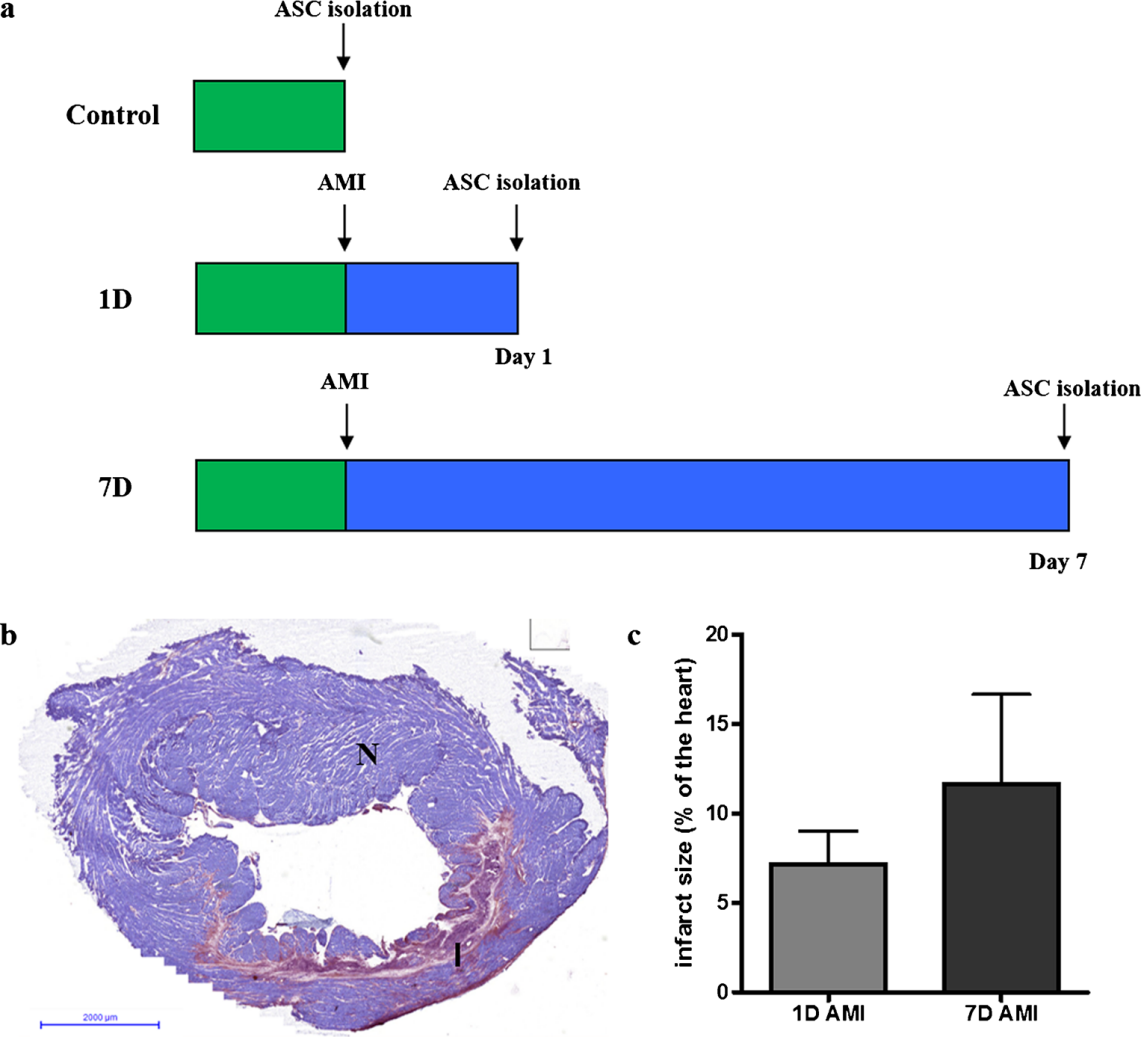
Experimental set-up and AMI. **a** Experimental set-up for the three different groups: *Control*, *1D* and *7D*. **b** Representative microscopical image of a PTAH staining to confirm infarction. PTAH stains viable cardiomyocytes purple (*N* for normal tissue), and damaged cells red/pink (*I* for infarcted tissue). **c** Infarct size as percentage of a heart slide. Infarct size of the 1D group was 7.2 ± 2.8 % and 11.7 ± 5.7 % in the 7D group. Data shown as mean ± SD (7D group *n* = 6, 1D group *n* = 5)

Acute myocardial infarction was induced in rats, whereafter adipose tissue was collected at day 1 (1D group, *n* = 5) or at day 7 (7D group, *n* = 6) post-AMI, followed by isolation of SVF cells (see Fig. 1a). As a control, SVF cells were isolated from healthy rats without AMI (Control group, *n* = 6). The success of the operational AMI procedure was determined by histological stainings for infarct size of heart slides with PTAH. For this, heart slides were evaluated distal from the suture placed on the left coronary artery descendent. A representative image of the PTAH staining of an infarction is shown in Fig. 1b. Infarct size of the 1D group was 7.2 ± 2.8 % of the myocardium, while the 7D group had an infarct size of 11.7 ± 5.7 % (Fig. 1c).

### Composition of the SVF after AMI

The SVF was analyzed for cell size, the percentage of ASC, and cell surface marker profile.

**Fig. 2:**
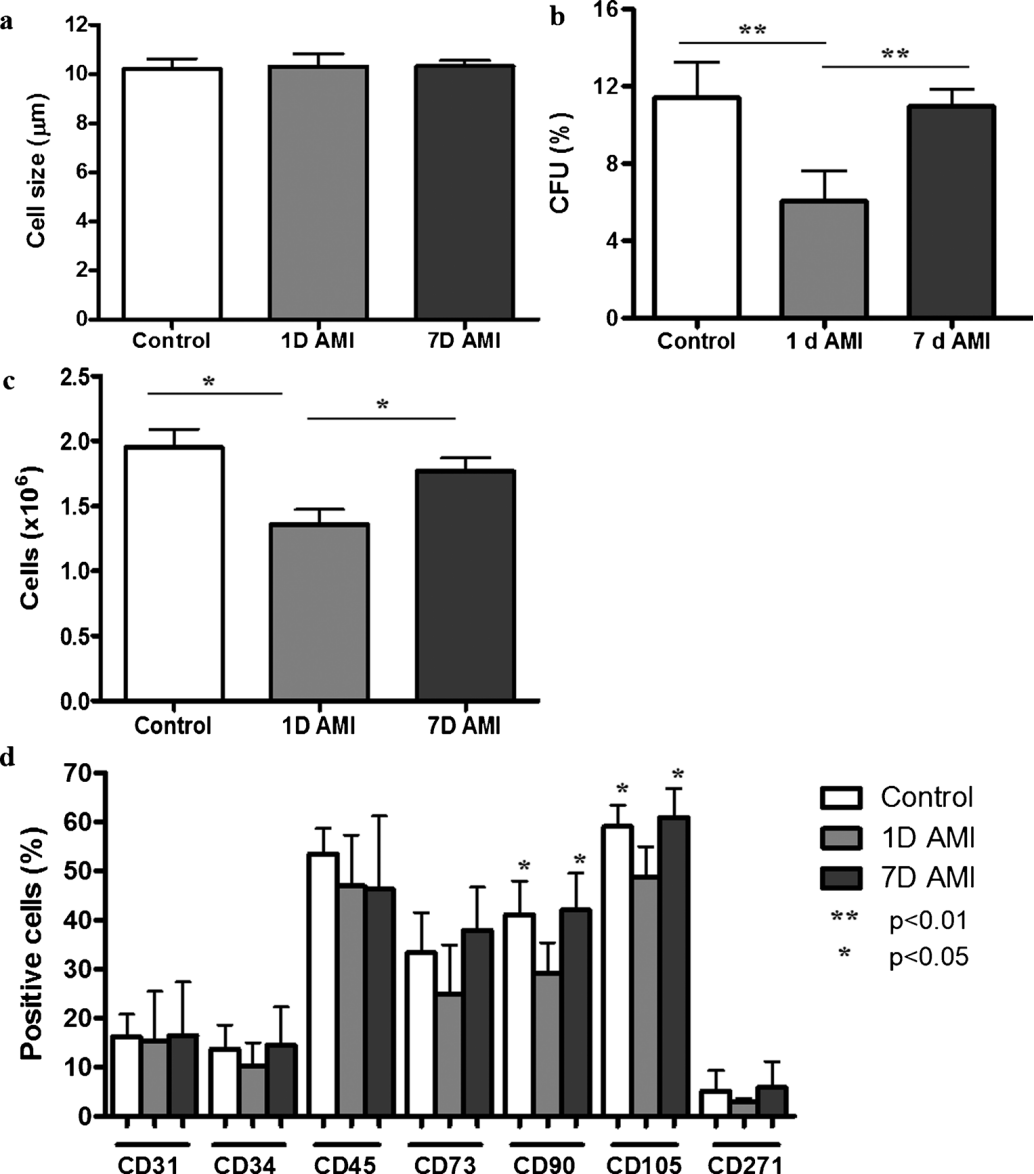
Effect of AMI on rat SVF cells. **a** Cell size (μm) of the SVF cells, demonstrating no difference between the three groups. **b** Percentage of colony-forming cells, showing significantly fewer colonies in the 1D group (6.1 ± 1.6 %) compared to the Control (11.4 ± 1.8 %) and 7D groups (11.0 ± 0.9 %). **c** Number of cells, obtained 4 days after seeding of the SVF fraction, demonstrating significantly fewer cells in the 1D group (1.3 ± 0.1 million cells) compared to the Control (2.0 ± 0.1 million cells) and 7D groups (1.8 ± 0.1 million cells). **d** Percentage of positive cells for the markers CD31, CD34, CD45, CD73, CD90, CD105 and CD271 showing significantly fewer CD90- and CD105-positive cells in the 1D group. Data shown as mean ± SD (Control and 7D group *n* = 6, 1D group *n* = 5, *p < 0.05, **p < 0.01)

No significant differences were found in average size of SVF cells directly after isolation between the different groups (Fig. 2a). To determine the percentage of ASC in the SVF fraction, a colony-forming-unit assay was performed (Bourin et al. 2013). The percentage of colony-forming cells, analyzed after 14 days of culture, was 11.4 ± 1.8 % in the Control group and 11.0 ± 0.9 % in the 7D group. Interestingly, in the 1D group, significantly fewer colonies were formed (6.1 ± 1.6 %, *p* < 0.01 compared with both groups), as shown in Fig. 2b. In addition, in the sham group, a similar number of colony-forming cells was found 1 day post-operation, as compared with the non-operated control rats. In line with this, culturing the SVF cells for 4 days resulted in significantly fewer cells in the 1D group (1.3 ± 0.1 million cells) compared with the Control group (2.0 ± 0.1 million cells, *p* < 0.05) and the 7D group (1.8 ± 0.1 million cells, *p* < 0.05), as depicted in Fig. 2c. Furthermore, the cell surface marker profile of the SVF cells showed significantly fewer CD90-positive cells in the 1D group (29.1 ± 6.3 %) compared with the Control group (41.1 ± 6.9 %, *p* < 0.05) and with the 7D group (42.1 ± 7.4 %, *p* < 0.05). In addition, significantly fewer CD105-positive cells were present in the 1D group (48.7 ± 6.3 %) compared with Control (59.1 ± 4.3 %, *p* < 0.05) and 7D groups (61.0 ± 5.9 %, *p* < 0.05). These results theoretically indicate that fewer ASC were present in the SVF of the 1D group. The same trend was observed for the percentage of positive cells in the 1D group for stem cell-associated markers CD34 (Control group 14.4 ± 5.1 %, 1D group 9.4 ± 4.9 %, 7D group 15.6 ± 7.8 %, *p* > 0.05), CD73 (Control group 33.3 ± 8.3 %, 1D group 24.8 ± 10.1 %, 7D group 37.9 ± 8.8 %, *p* > 0.05) and CD271 (Control group 5.1 ± 4.1 %, 1D group 2.8 ± 0.8 %, 7D group 5.9 ± 5.2 %, *p* > 0.05) (Fig. 2d). No change was found for the percentage of CD31-positive cells, an endothelial cell marker (Control group 16.2 ± 4.8 %, 1D group 15.2 ± 10.3 %, 7D group 16.4 ± 10.9 %, *p* > 0.05), or for the percentage of CD45-positive cells, a marker for leukocytes (Control group 53.5 ± 5.2 %, 1D group 46.9 ± 10.4 %, 7D group 46.4 ± 14.8 %, *p* > 0.05). These results show changes in cellular composition of the SVF 1 day after AMI, with less ASC in the SVF in the 1D group. Sham-operated rats showed similar cell surface markers as non-operated control rats (not shown).

### Effect of AMI on functional characteristics of ASC

**Fig. 3:**
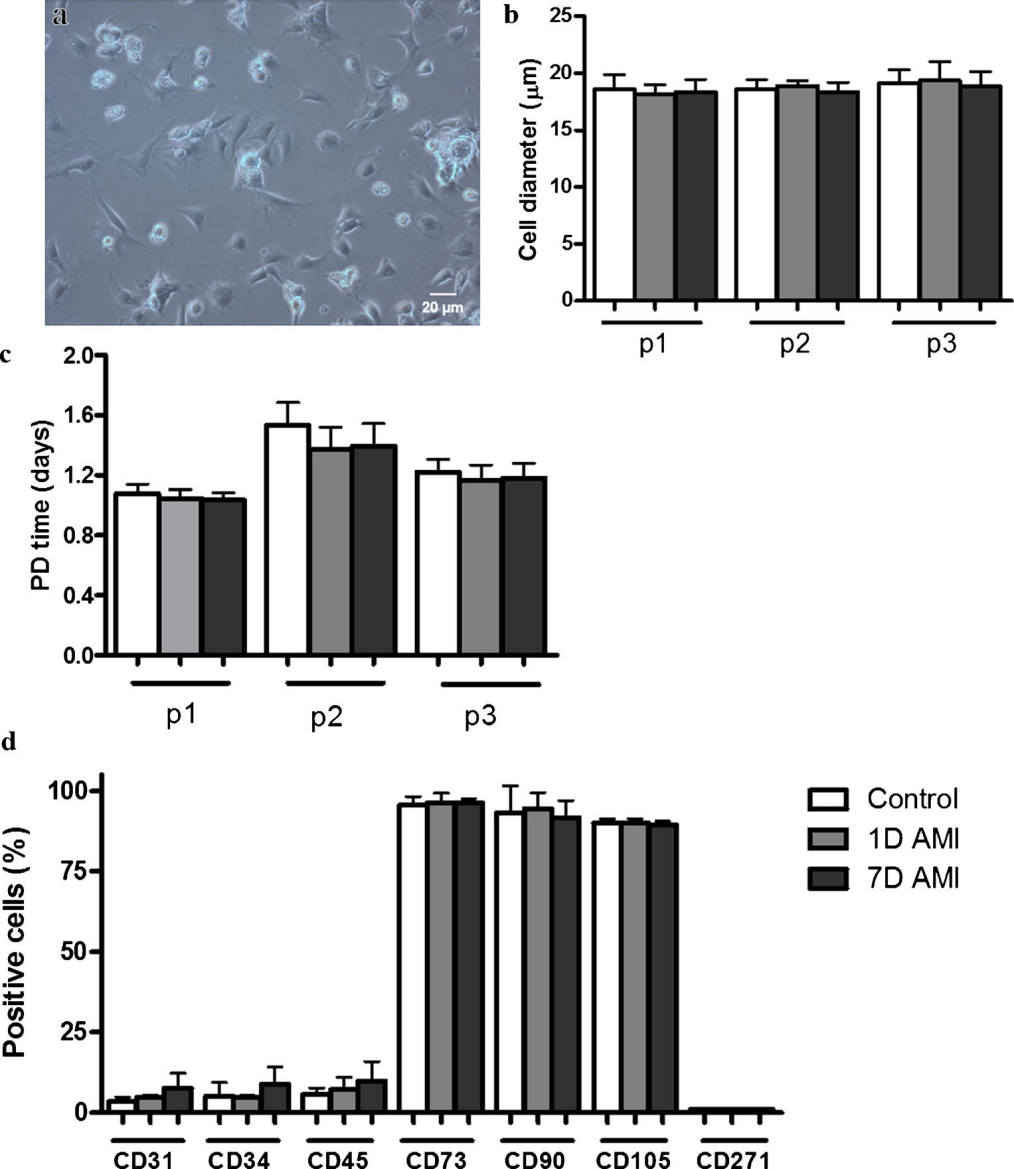
Effect of AMI on cultured rat ASC. **a** Representative light microscopy image demonstrating the morphology of cultured rat ASC. **b** Cell size (μm) of ASC, demonstrating no significant differences between the three groups. **c** Population doubling time (in days) depicted for passages 1–3, showing no significant differences between the three groups. **d** Percentage of positive cells for the markers CD31, CD34, CD45, CD73, CD90, CD105 and CD271, showing no significant differences in numbers of positive cells between the groups. Data shown as mean ± SD (Control and 7D group *n* = 6, 1D group *n* = 5)

After culturing the heterogenous SVF in stem cell proliferation medium, a more homogenous ASC population was obtained. Characteristics of these cultured ASC, such as morphology, proliferation, cell size and surface marker profile, were determined. ASC from both AMI groups showed similar morphology and this was no different from the Control group (representative image shown in Fig. 3a). ASC were cultured for three passages, during which no differences in average cell size were found between the groups (Fig. 3b), nor in the population doubling time (Fig. 3c). Furthermore, cell surface marker profiles of ASC, analyzed in passage 2, did not differ between the three groups (Fig. 3d), showing a normal rat ASC surface marker profile (van Dijk et al. 2011). Thus, the ASC cultured from the SVF of all groups showed similar culture characteristics.

### Differentiation capacity after AMI

**Fig. 4:**
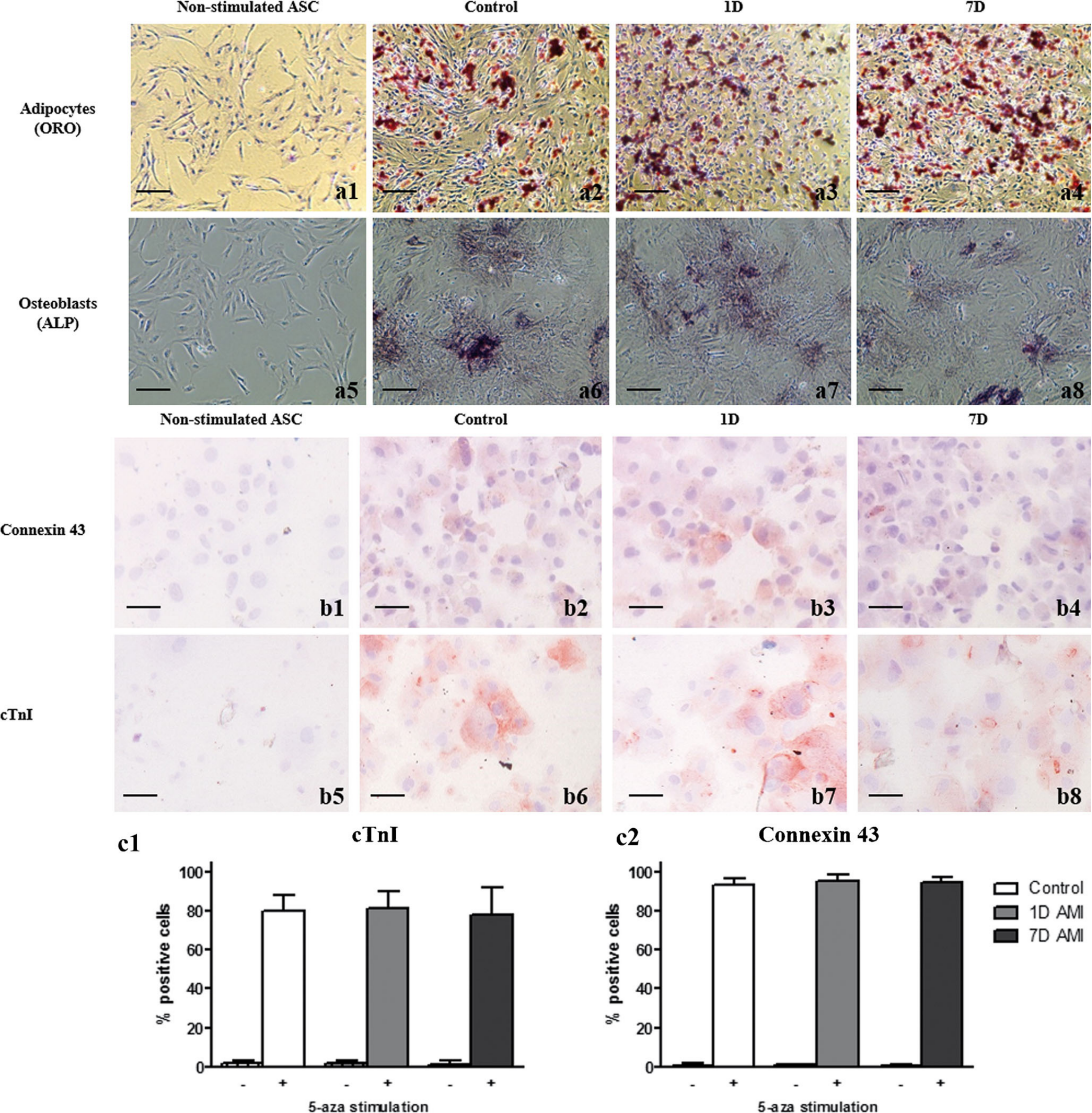
Multilineage differentation of rat ASC. (**a**) Representative images showing differentiation of ASC towards osteoblasts, shown by ALP staining, and adipocytes, shown by Oil Red O staining (a1-a4). No staining is found in the non-stimulated cells (a5-a8). (**b**) Representative images of differentiation towards cardiomyocytes, staining for connexin 43 (*upper panel*, b1-b4) and cTnI (*lower panel*, b5-b8) on cytospin slides. (**c**) Bar graph showing quantification of the number of positive ASC for cTnI (c1) and connexin 43 (c2) without (−) or stimulated (+) with 5-aza-2-deoxycytidin. No significant differences in differentiation between the three groups were found. Data shown as mean ± SD (Control and 7D group *n* = 6, 1D group *n* = 5)

It was subsequently investigated whether AMI affected the differentiation capacity of ASC in culture passage 1. To show differentiation of the ASC towards adipocytes and osteoblasts, ASC were stimulated with adipogenic and osteogenic differentiation medium, for 7 and 12 days, respectively. For both differentiation assays, non-stimulated ASC were added as controls. In Fig. 4a, representative images of the differentiation assays are shown, showing ALP staining for the stimulated cells from Control, 1D and 7D groups, but not for the non-stimulated ASC. In addition, lipid vesicles were present in the stimulated cells from Control, 1D and 7D groups, but not in the non-stimulated ASC. This indicates that all three groups were still capable of differentiating towards osteoblasts and adipocytes, respectively. Differentiation towards cardiomyocytes was induced by stimulation with 5-aza-2-deoxycytidine as described previously by van Dijk et al. (2008). Microscopy images showed that the stimulated ASC expressed connexin 43 and cTnI, markers of cardiomyocytes (Naaijkens et al. 2012), while non-stimulated ASC did not (Fig. 4b). No difference in the percentage of differentiated ASC between all three groups was found for connexin 43 (Control group 91.4 ± 3.3 %, 1D group 93.6 ± 2.7 %, 7D group 93.7 ± 1.9 %; Fig. 4c) and cTnI (Control group 79.8 ± 8.2 %, 1D group 81.4 ± 8.7 %, 7D group 78.0 ± 13.8 %; Fig. 4c). As such, ASC from both healthy control rats as well as from rats that suffered AMI were capable of differentiating towards adipocytes, osteoblasts, and, most importantly, towards cardiomyocytes.

## Discussion

In clinical practice patients that suffered from AMI undergoing stem cell therapy will receive autologous stem cells. However, it is unknown whether AMI affects the patients’ own stem cells. In the present rat study we analyzed the putative effect of an AMI on ASC. We found that significantly fewer ASC were present in the adipose tissue isolated one day post-AMI compared with healthy controls and seven days post-AMI, shown by a colony forming unit assay and cell surface marker profile of the SVF cells. Sham operations confirmed that the decrease in number of ASC was due to the AMI, and not due to the operation. After culturing, ASC from the three groups showed no differences in culture characteristics, including cell size, proliferation rate or differentiation capacity. Thus, AMI affected the number of stem cells in the adipose tissue, but not their functional characteristics.

A possible explanation for the decrease in the number of ASC could be that upon AMI they were mobilized into the peripheral blood. This is in agreement with a number of studies that demonstrated a significant upregulation in the number of stem cells in peripheral blood four days post-AMI in mice (Dutta et al. 2012) and in patients one day post-AMI (Wojakowski et al. 2006). In addition, the study of Iso et al. (2012) described a significant increase in circulating mesenchymal stem cells in blood of patients three days after AMI, compared to day zero and seven post-AMI, which is in agreement with our rat study. These three studies suggested that stem cells were mobilized from the bone marrow, assuming all mesenchymal stem cells originate from the bone marrow. Now our results suggest that not only the bone marrow, but also the adipose tissue may release stem cells after AMI, which has to the best of our knowledge not been shown before. No distinction between ASC or bone marrow mesenchymal stem cells can be made based solely on cell surface markers, therefore we did not determine the number of circulating ASC in peripheral blood. Another explanation for the lower number of ASC present in the SVF one day after infarction might be that ASC in the adipose tissue died one day post-AMI due to the AMI-induced systemic inflammatory response (Krijnen et al. 2006). However, this is unlikely, since stem cells are able to resist hostile environments (van Dijk et al. 2012). Furthermore, we found that seven days post-AMI, the percentage of ASC in the adipose tissue was again similar to that in healthy controls. This might be due to proliferation of the ASC that remained in the adipose tissue, or differentiation of early progenitor cells into ASC, to replenish the depleted cells.

No other studies have investigated purely the effect of AMI on ASC. However, there are other studies which have analyzed the putative effects of aging or obesity on ASC. It has been shown in vitro that aging negatively affected the proliferation rate of human ASC, as well as the angiogenic stimulatory capacity in tube-forming assays and differentiation towards osteoblasts (Efimenko et al. 2011; Madonna et al. 2011). It has also been shown that obesity significantly lowered the number of CD90-positive cells in the SVF fraction of human obese patients, similar to the effect of AMI on CD90-positive cells in our rat study. However, obesity also negatively affected ASC in proliferation and differentiation capacity towards adipocytes (Onate et al. 2012). Of course, obesity is a long-term disease that develops in patients over a period of several decades, in contrast to an acute myocardial infarction induced in rats. This may explain the differences of ASC concerning proliferation rate and differentiation capacity. The percentage of patients that suffered AMI and also had obesity is 28.9 %, a high incidence (Oda et al. 2012). Thus, for the clinical situation, obesity might negatively affect ASC therapy post-AMI.

In this study, it was shown that cultured ASC had the same properties when isolated after AMI compared with healthy controls. Proliferation rate as well as cell surface marker profile and differentiation capacity towards cardiomyocytes of rat ASC from the different groups were comparable to what has been described previously for rat ASC (van Dijk et al. 2011; Carvalho et al. 2013), suggesting that AMI did not affect the functional characteristics of ASC. Furthermore, it was found that the infarct size in the 1D group was lower compared to the 7D group, although this was not a significant difference. This increase in infarct size can be explained by the post-AMI induced inflammatory response, subsequently jeopardizing cardiomyocytes (Krijnen et al. 2006; van Dijk et al. 2009).

In conclusion, we showed in rats that AMI affected the composition of the SVF 1 day post-AMI via a decrease in the number of ASC; however, the ASC that were present showed no functional differences. Therefore, we hypothesize that stem cells can still be isolated and applied 1 day post-AMI without having any impairments in the functional characteristics of ASC, including differentiation to cardiomyocytes when applied as regenerative therapy. However, this should be further investigated in future experiments to determine whether these ASC also show a similar reparative capacity in vivo. Fortunately, adipose tissue is a rich source of mesenchymal stem cells, providing sufficient stem cells for cellular therapies at any time point of isolation.

## Abbreviations

AMI: Acute myocardial infarction
ASC: Adipose-derived stem cell
SVF: Stromal vascular fraction
